# Between Public Guidelines for User Involvement and Ideals About Free Research: Using Collaborative Autoethnography to Explore Researcher Experiences From a User Involvement Process

**DOI:** 10.1111/hex.70055

**Published:** 2024-10-09

**Authors:** Anne Werner, Ingrid Ruud Knutsen, Anne‐Kari Johannessen

**Affiliations:** ^1^ HØKH—Health Services Research Unit Akershus University Hospital (Ahus) Lørenskog Norway; ^2^ Department of Nursing and Health Promotion Faculty of Health Sciences, OsloMet—Oslo Metropolitan University Oslo Norway

**Keywords:** collaborative autoethnography, James Lind Alliance, reflexivity, researcher perspective, user involvment

## Abstract

**Background and Aim:**

User participation is a prerequisite for receiving research funding in healthcare in Norway. Despite many positive benefits, studies report challenges from users' and researchers' perspectives. Limited knowledge exists concerning researchers' experiences in scenarios where the users are professionals within healthcare and research. The aim of this retrospective study was to explore and reflect on personal experiences as researchers from a process of planning and developing research questions for a PhD project, following the James Lind Alliance guidelines, which were a requirement for funding. We focused on how the process of collaboration with a specific group of users influenced the researchers' sense of selves.

**Design and Method:**

We used a qualitative design based on collaborative autoethnography, exploring personal experiences from a sociocultural point of view. Two of the three researchers in the team recollected their experiences from the user involvement process while applying the James Lind Alliance guidelines. We used different data sources to develop two autoethnographic narratives. The narratives were analysed using thematic analysis.

**Results:**

The autoethnographic narratives demonstrate the complexity of user involvement from the researchers' perspectives. We identified four themes in the analysis: intrinsic and extrinsic motivation, competing paradigms, hierarchy and dual roles. The accounts illustrated the researchers' ambivalence within the process, indicating that they feared a loss of control over the direction of the research project. The narratives visualised a struggle to appear as credible researchers, illustrating how the involvement of a specific group of users and adherence to a specific guideline for user involvement influenced the researchers' experiences of their roles and identities in the collaboration.

**Conclusion:**

The results point to the relevance of the sociocultural backdrop; researchers might become frontline providers of policy implementation in research, balancing tensions between regulatory constraints, user involvement and researchers' professional identity and research ideals, when a specific, detailed procedure for user involvement is required.

**Patient or Public Contribution:**

Two user panels comprising participants from clinical practice, education and research, along with a service user, collaborated in the planning and development of research questions for a PhD project. This autoethnographic study elaborates this process.

## 1 Introduction

### 1.1 User Participation in Healthcare Research

User participation has been a legal right in healthcare services in Norway for several years [1]. In recent decades, there has been an international trend to request user participation in the research process, with the active involvement of patients and the public, who are the focus of the research [2]. Norway has followed this development in its policy guidelines and calls for proposals, implying that if user participation is not included in the research, a justification must be provided [1]. The objective is to enhance the quality and relevance of research for clinical practice and healthcare services, thereby empowering patients and caregivers [1, 3]. The concept of users often refers to patient and public involvement, including carers. The Research Council of Norway also includes healthcare professionals, service leaders, organisers and decision‐makers [4].

Various concepts are used in the literature to conceptualise the participation of users, such as user participants, user involvement, patient and public involvement or co‐production in research [1, 5]. User involvement is defined as a partnership between researchers and users in some or all steps of a project [1]. A co‐researcher is a fully included peer member of the team, beyond the traditional role of users as advisers. However, in the literature, ‘user involvement’ is a concept with multiple definitions, often used inconsistently and synonymously with ‘user participation’ [6]. In this study, we vary between ‘user participation’ and ‘user involvement’, depending on the context to which we refer.

Some researchers have expressed concern regarding whether user participation comes at the cost of more advanced knowledge production [1, 7, 8]. During the last 15 years, there has been a focus in articles that a ‘disturbingly’ large part of healthcare research is ‘wasted’ [9, 10, 11]. This is defined as ‘choosing the wrong question for research’, ‘doing research that is unnecessary or poorly designed’, ‘unusable research’ or ‘failure to publishing or implementing relevant research’ [9]. It also points out that ‘waste’ is caused by potential neglect of users' needs [10] and a mismatch between the agendas of users and the researchers [5].

Diverse guidelines for involving users in research have been developed, including the James Lind Alliance (JLA) method for involving patients, carers and clinicians in setting research priorities, which aims to counter these challenges [12]. Following the guidelines involves bringing these parties together by establishing Priority Setting Partnerships (PSP). The participants work together to identify and prioritise ‘evidence or treatment uncertainties'—that is, unanswered research questions—to develop a top 10 priority list of research needs that patients, carers and clinicians agree on as topics that ‘matter most’ and deserve ‘priority attention’ [12].

**Figure 1:**
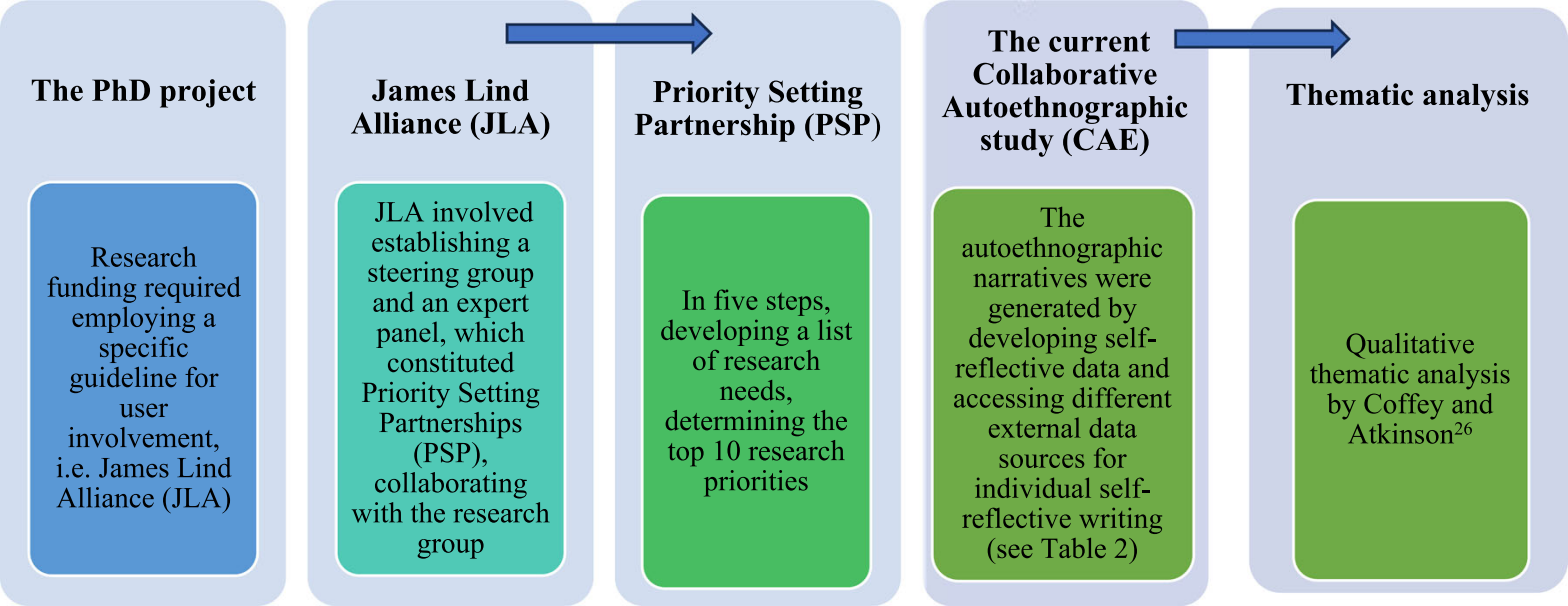
Use of concepts and the connection between the concepts and the current retrospective study.

Studies report varied experiences from collaborations with users, highlighting challenges from both users' and researchers' perspectives [1, 13, 14]. Andreassen argues that models or measures designed to strengthen the position of service users not only alter their position but also the position of the professionals involved [15]. Knowledge is scarce regarding processes in which participating users are mainly expert providers by being professionals in healthcare and research. Furthermore, knowledge about researchers' perspectives, specifically regarding the adoption of specific method for user involvement, is limited. Given the considerable emphasis on requirements for user involvement in research policy, we believe there is a need for increased knowledge on how such processes are experienced from researchers' perspectives and how this involvement may possibly influence their position. In this study, we draw on experiences from a PhD project that entailed an active involvement of users in developing research questions. Figure 1 illustrates how this current retrospective study relates to the PhD project, which included following the JLA.

### 1.2 Writing the Self Into Research in a JLA Project

When seeking funding for a PhD project some years ago, a precondition in the call was to perform the study in accordance with or inspired by the JLA guidelines [12]. As a supervisor team of three established researchers employed in the university sector, we aimed to succeed in achieving funding and adhering the project to the JLA. Following this standard, we established two user panels for PSP for the PhD project: a steering group and an expert panel. With the expert panel, we discussed research topics that we had identified and prioritised in collaboration with the steering group. Due to the focus of the PhD project, health professionals and leaders from clinical practice and research were invited to the user panels, along with a former patient (Table 1).

**Table 1:** James Lind Alliance: the user participants who constituted the Priority Setting Partnership in the PhD project.

User participant representatives	The steering group (*N* = 10 user participants)	The expert panel (*N* = 5 user participants)
Health professionals from various hospital departments and education	Two Senior Physicians, MD with PhD, Anaesthesia and Neurology One Senior Physician, MD, Anaesthesia One Section Leader, RN, Gastrology One Special Nursing Advisor, RN, Simulation Training Three RNs, Professional Development Coordinator for nursing staff One RN, Orthopaedics	One Head of Department, MD with PhD One Section Leader, RN, Intensive Care Unit One Senior Physician, MD, Anaesthesia One Special Advisor, RN, Simulation Training One Associated Professor, RN, Nursing University
Service user	One user participant from an organisation of patients and carers	
Organisers of the James Lind Alliance (JLA) Priority Setting Partnership (PSP)	The PhD fellow The supervisor team of three researchers	The PhD fellow The supervisor team of three researchers

Abbreviations: RN = registered nurse, MD = medical doctor.

In the JLA guidelines, the process of developing a priority list of research needs is expected to take 18 months [12]. Given the time limit of a PhD project, we conducted three meetings over a 6‐month period, developing a pragmatic approach to the five stages of JLA PSP: (1) setting up a PSP, (2) gathering uncertainties, (3) assembling potential research questions, (4) ranking of treatment ‘uncertainties' and (5) determining the top 10 research priorities [16].

The JLA PSP has also been called ‘needs‐led research’ [17]. Following the JLA implied a more genuine and extensive collaboration between users and researchers than we were used to [12]. In previous research, we have applied general Norwegian policy guidelines for user participation [4]. These guidelines imply that we have invited users in the project's advisory board to give advice in different phases according to research questions developed in advance. The JLA prescribes a more detailed procedure for how to proceed, inviting patients, carers and clinicians to identify unanswered research questions and prioritise between them [12].

During the process, we often discussed experiences in the research team from our collaboration with the specific user groups, following the JLA method, which we experienced as a strict approach. In this study, our experiences of user involvement in the JLA process were the point of departure. Coming from different professions and research backgrounds, we wanted to retrospectively explore our experiences from a broader sociocultural perspective.

### 1.3 Aim

The aim of this retrospective study was to explore and reflect on personal and professional experiences as researchers from a process of planning and developing research questions for a PhD project, following the JLA guidelines. We focused on how the process of collaboration with a specific group of users influenced the researchers' sense of selves.

### 1.4 The Dilemmas of the Street‐Level Bureaucrats—A Theoretical Perspective

Michael Lipsky has described, from a public policy discipline, how different kinds of frontline providers work within a paradoxical framework, with conflicting and ambiguous goals in public service [18]. His perspective on the ‘street‐level bureaucracy’ has been used in several studies to illuminate different kinds of frontline providers' challenges in their interaction with service users [19, 20, 21]. Their responsibilities are often marked by tensions that arise from balancing users' needs, available resources and regulatory constraints. Focusing on how providers manage these dilemmas, Lipsky argues that ‘street‐level bureaucrats' act as liaisons between policymakers and citizens, trying to find acceptable solutions that harmonise with these decisions [18]. Lipsky's perspective on the dilemmas of frontline providers sheds light on what could be at stake in the user involvement process from the researchers' perspectives.

## 2 Qualitative Design

### 2.1 Collaborative Autoethnography (CAE) as a Method

We applied a qualitative research design, drawing on CAE outlined by Chang, Hernandez, and Ngunjiri [22]. Autoethnography (AE) is a method that uses personal memories as empirical data to describe and systematically analyse recollections, aiming for an understanding of sociocultural aspects of experiences [22, 23]. AE is a solo performance in the exploration of self [22]. In CAE, researchers work as a collaborative ensemble, collecting, analysing and interpreting the autoethnographic data to create a shared understanding of sociocultural phenomena. During the process, the researchers alternate between group and solo work [22, 24, 25]. The purpose is to challenge notions and practices, which are often taken for granted, by critically examining cultural, social and political conditions through a lens of personal experiences. We aimed to use our self‐accounts from the user involvement process as ‘a window to society’, as expressed by Chang, Hernandez, and Ngunjiri [22].

### 2.2 Data Collection and Data Analysis

We used a three‐step guideline of AE (Table 2) [23].

**Table 2:** A three‐step guideline of autoethnography [23].

The three‐step guideline	The autoethnographic study
1. **Getting started—the research question:** In composing the research question, it is important that it reflects the focus of autoethnography, which explores a cultural issue through one's personal story.	The aim of the retrospective study was to explore and reflect on personal and professional experiences as researchers from a process of planning and developing research questions for a PhD project, following the James Lind Alliance guidelines, which was a requirement for funding. We focused on how the process of collaboration with a specific group of users influenced the researchers' sense of selves.
2. **Data collection:** a Because your own life is the primary source of information, there are several ways to collect your data, for instance: a **Developing self‐reflective data**, journaling your reflections about your experience and perceptions related to the topic and asking yourself a series of questions related to the topic. b **Access to external data**, such as photographs, letters, diaries, reports, and other documents or artefacts that are pertinent to your study.	For **developing the autoethnographic narratives**, we collected different data to contribute to self‐reflective writing: We developed self‐reflective data, journaling our reflections about our experiences and perceptions related to the topic, recollecting memories of experiences from the JLA process and asking ourselves a series of questions: How did user involvement in planning and developing research questions for the PhD project following the JLA impact me as a researcher and my overall sense of self? What influence did followin\g JLA have on my understanding of myself as a researcher? What makes, or what has made me the researcher I am? We accessed different kinds of **external data**: **Written reports** from: The three meetings with the user panels in the PhD project: two meetings with the steering group and one meeting with the expert panel. Participating in one of the seminars arranged by the PhD funder on topics including the JLA method. **Written personal logs** of reflections on experiences and feelings from: The three meetings with the user panels. Participating in four of the seminars arranged by the PhD funder on the JLA method. Articles and publications about ‘wasted research’, which is part of the background for the JLA guidelines and the research funding^b^: Chalmers I, Glasziou P. Avoidable waste in the production and reporting of research evidence. *The Lancet*. 2009;374(9683):86‐89. https://doi.org/10.1016/S0140-6736(09)60329‐9 Chalmers I, Bracken MB, Djulbegovic B, et al. How to increase value and reduce waste when research priorities are set. *The Lancet*. 2014;383(9912):156‐165. https://doi.org/10.1016/S0140-6736(13)62229‐1 Yordanov Y, Dechartres A, Porcher R, Boutron I, Altman DG, Ravaud P. Avoidable waste of research related to inadequate methods in clinical trials. *BMJ*. 2015;350:h809. 10.1136/bmj.h809 James Lind Alliance. JLA Guidebook [Version 10]. March 2021. Accessed December 19, 2023, JLA Guidebook | James Lind Alliance (nihr. ac. uk)
3 **Data analysis** a: In analysing your data for your autoethnographic study, you can draw upon several qualitative traditions, for example, **general, descriptive qualitative research,** using descriptive coding.	For analysing the two autoethnographic narratives, we used a **qualitative method for coding and interpretation**—a **thematic analysis** outlined by Coffey and Atkinson [26].

*Note:* The bold text highlights key points in the guideline and what we have done in this study.

a Data collection and data analysis are not linear processes but a back‐and‐forth process.

b These external data sources of publications correspond to references [9, 10, 11, 12].

The first step was to compose our research question. We wanted to address the cultural issues in the researcher accounts from collaborating with users in the JLA while developing research questions for the PhD project [23]. In CAE, this step involves pre‐dialogues of discussions, deciding on a topic for individual self‐writing and primary data collection [22, 25]. The first and last authors were in charge of carrying out the JLA process in collaboration with the PhD fellow. Therefore, we agreed to write two individual stories.

The second step was data collection [23]. For developing the two autoethnographic narratives, we collected different kinds of data, both self‐reflective data and ‘external data sources', to contribute to the self‐reflective writing of recollections. We reread reports and logs from meetings and literature on ‘wasted research’ and JLA [9, 10, 11, 12]. We also asked ourselves a series of questions inspired by an autoethnographic study on cancer [24]:

- How did user involvement in planning and developing research questions in the PhD project following the JLA impact on me as a researcher and my overall sense of self?
- What influence did following JLA have on my understanding of myself as a researcher?
- What makes or what has made me the researcher I am?

In CAE, the second step involves both individual self‐reflective writing and collaborative dialogues [22, 25]. Drafts are shared and discussed, followed by additional self‐reflective writing and subsequent data collection. All three researchers routinely met to discuss written drafts of the two narratives, alternating between group and solo work. During this process, new perspectives were added to the drafts. The second author commented from a more distant position, suggesting adding our educational backgrounds, twisting parts of the draft in other ways and balancing the text more effectively.

The third step was the data analysis of the narratives to systematically analyse recollections [23]. In CAE, this step is called post‐dialogues, involving finalising the self‐reflective writing, generating the autoethnographic narratives and coding and interpreting the narratives [22, 25]. The autoethnographic narratives constituted both the results and data in this study [23]. We followed the principles of thematic analysis of qualitative data outlined by Coffey and Atkinson [26]. They describe the process of generating codes and themes as a mixture of data reduction and data complication. By examining the two narratives in the data reduction phase, we identified patterns of similarities and differences in the reflections from the user involvement process in relation to ambivalence. The data complication phase included linking the data to theory. Lipsky's perspective offered a lens to understand the narratives from the JLA process as parallels to the dilemmas of frontline providers [18]. In the analysis, we focused on challenges or ambivalences being expressed in the narratives in relation to the researchers' role.

## 3 Results

In the following subsections, we present the two researcher narratives of experiences and reflections, and the interpretation of these narratives based on thematic analysis.

### 3.1 The Autoethnographic Researcher Narratives

#### 3.1.1 A Medical Sociologist's Researcher Account—Teetering Between Feelings of Frustration, Insecurity and Pride

To describe my experiences with the user involvement process, following the JLA guidelines, I will step back to my educational background. More than two decades ago, I was trained in sociology, specialising in health and illness, qualitative methods and gender perspectives on theory and methodology. This implied taking a critical perspective on how knowledge of gender and society had been produced within sociology, criticising it for often adopting a top‐down perspective *about* people and their lives from an outside researcher's perspective instead of taking a bottom‐up perspective *for* people, mapping what is important to their lives from *within* and *below* [27, 28, 29].

While planning and preparing for the PSP of JLA, I participated in seminars arranged by the funder, aiming for an understanding of what it meant to perform needs‐led research. I struggled to understand how this guideline related to critical methodological perspectives embedded within social sciences and gender studies during the 1980s and 1990s. Specifically, I questioned the methodological differences or similarities between critical approaches, such as ‘participatory research’ or other research methods developed to enhance and involve the voices of those engaged in the study. I was left questioning whether there was a lack of historical knowledge behind the establishment of the JLA.

As I read articles in highly ranked scientific journals about the need ‘to avoid wasted research’ [9, 10, 11], I felt a sense of embarrassment recalling my thesis from many years ago. I had aimed to contribute to better healthcare for women with chronic pain. In retrospect, I wondered whether the developed knowledge might not have been perceived as useful by either the patients or the general practitioners [30, 31]. I also rendered myself suspected as a researcher, wondering whether words like ‘wasted research’ could be used to characterise my studies.

I found myself teetering between feelings of insecurity and frustration when planning meetings with the user panels. I felt frustrated regarding the necessity to follow JLA standards and uncertain about how to pragmatically use this process and comprehend the implications. Despite taking pride in my background in social science and being equipped with a toolbox of methods and theories, I was insecure when meeting with the user participants of professionals possessing extensive expertise from clinical practice in the field of the PhD project, which I lacked. Still, I wondered whether ‘hunting for’ research questions were possible within these meetings. Would it be possible without us researchers doing a kind of ‘legwork’ before the meetings? I also wondered whether several meetings with the user panels were necessary to develop research questions for the project. I was convinced that as a research team, we needed to govern the process, not least to uphold progress in the PhD project. At the same time, I struggled with feelings of being undervalued or not recognised as a researcher when the PhD fellow had to undergo training in the JLA method by the funder.

#### 3.1.2 A Nurse University Teacher's Researcher Account—**Teetering** Between Feelings of Excitement, Inferiority and Insecurity

My professional background as an anaesthetist nurse, coupled with decades spent working in hospitals, has significantly shaped my perceptions of how healthcare professionals approach and practice user participation. I have observed that the mindset of hospital professionals often reflects traditional medical science, characterised by paternalistic tendencies. I believe this inclination is natural and frequently unconscious, driven by good intentions.

Hence, I was genuinely enthusiastic and grateful for the opportunity to employ the JLA method alongside my research colleagues and the engaged user panels to enhance the democratisation of research. In my view, clear principles for user participation were necessary, as JLA guidelines encompass such involvement. At the same time, I felt insecure about this because I had had no experience with this method during my 10 years as a researcher. Furthermore, I wondered how well the user panels would adapt to this collaborative approach alongside our research team.

Within the participating panels, several members possessed more extensive research experience and subject expertise than our research team. This dynamic made me feel like I was walking a fine line during our meetings, recognising their deeper knowledge within the clinical field. However, my professional background as an anaesthetist nurse granted me a positive sense of belonging and affiliation with this group, as I shared their clinical language and modes of reasoning. Nonetheless, engaging with them on their own turf necessitated thorough preparation before meetings to maintain trust and respect, especially in terms of showcasing my research qualifications and standards. I felt a strong need for security and control during these unfamiliar encounters. Questions such as ‘What could I contribute as a researcher to the user involvement process?’ and ‘How could I quickly gain their acceptance and acknowledge the value of their input?’ crossed my mind. It was as though there was a silent exchange of ‘Who owes what to whom?’ or ‘What are our mutual obligations?’ This was further complicated by the question of whether using their time would entail future reciprocity.

As a researcher, I recognised the importance of accepting and navigating power dynamics within the team, knowing that this collective effort would ultimately refine the research questions for the PhD project. Being pragmatic, responsive, attentive and appreciative towards other participants was crucial in defining my role. In hindsight, I consider this phase of the research to be both personally challenging and educational, and it undoubtedly contributed to the success of the PhD project.

### 3.2 Interpretation of the Autoethnographic Narratives

The two autoethnographic narratives demonstrate the complexity of user involvement from the researchers' perspectives. The narratives have different angles as points of departure for carrying out JLA and offer reflections on the process leading up to performing JLA, illuminating an ambivalence in different ways. The accounts visualised varying feelings from excitement, pride and frustration to insecurity, inferiority and vulnerability, illuminating a fear of losing control of the direction of the project. The narratives illuminated various tensions embedded in the collaboration with the users and performing user involvement according to the JLA. The narratives indicated how the involvement of a specific group of users, following the specific guideline for user involvement, influenced the researchers' experiences of their role and identity in the collaboration, and a struggle to appear as credible researchers in the process.

In the analysis of the narratives, we identified four themes, which are taken further in the discussion: intrinsic or extrinsic motivation, competing paradigms, hierarchy and dual roles. Table 3 presents examples of the analysis.

**Table 3:** Examples of the thematic analysis process [26].

	The autoethnographic narratives—parts of the written texts	Codes— categories	Themes
3.1.1 A medical sociologist's researcher account	I felt frustrated regarding the necessity to follow the JLA guideline and uncertain about how to pragmatically use it and comprehend the implications. Despite taking pride in my background in social science and being equipped with a toolbox of methods and theories, I was insecure when meeting with the user participants of professionals possessing extensive expertise from clinical practice. Still, I wondered whether ‘hunting for’ research questions were possible within these meetings. Would it be possible without us researchers doing a kind of ‘legwork’ before the meetings? … I was convinced that as a research team, we needed to govern the process, not least to uphold progression in the PhD project. At the same time, I struggled with feelings of being undervalued or not recognised as a researcher when the PhD fellow had to undergo training in the JLA method by the funder.	Ambivalence—ambivalent feelings: *Feelings of insecurity when meeting with the user panels of professionals possessing extensive expertise from clinical practice* *Feelings of insecurity and frustration when planning meetings with user participants*	(Extrinsic) Motivation Competing research paradigms Dual roles Hierarchy
3.1.2 A nurse university teacher's researcher account	I have observed that the mindset of hospital professionals often reflects traditional medical science, characterised by paternalistic tendencies. Hence, I was genuinely enthusiastic and grateful for the opportunity to employ the JLA method alongside my research colleagues and the engaged user panels to enhance the democratisation of research. In my view, clear principles for user participation were necessary, as JLA guidelines encompass such involvement. At the same time, I felt insecure about this because I had had no experience with this method during my 10 years as a researcher. Furthermore, I wondered how well the user panels would adapt to this collaborative approach alongside our research team. Within the participating panels, several members possessed more extensive research experience and subject expertise than our research team. This dynamic made me feel like I was walking a fine line during our meetings, recognising their deeper knowledge within the clinical field.	Ambivalence—ambivalent feelings: *A positive sense of belonging and affiliation to the clinical group* *Feelings of insecurity due to the lack of experience with the JLA method* *Recognising the user participants' deep knowledge within the clinical field*	Competing research paradigms (Intrinsic) Motivation Dual roles Hierarchy

## 4 Discussion

We are not the first to report varied experiences and challenges in user involvement processes in research [1, 13, 14]. The literature on this topic is considerable. However, although varied experiences from both users' and researchers' perspectives have been reported [13], the focus has predominantly been on the benefits for users [32]. Some research communicates certain negative co‐impacts experienced by users, linking them to emotions such as not being heard, insecurity, distress, lack of control and powerlessness [33, 34, 35]. Interestingly, some of these experiences are similar to those unveiled in the two narratives. Numerous studies indicate that negative experiences among users are often attributed to the behaviour and attitudes of researchers who are commonly perceived as disinterested or unwilling to collaborate [36, 37, 38]. A recent shift in focus has increased attention directed towards understanding researchers' perspectives [39, 40]. Our study contributes to existing knowledge by presenting insights into researchers' experiences and reflections from ‘within’ a user involvement process in a healthcare study, where users are predominantly healthcare professionals or researchers themselves and, thus, more equally positioned. The narratives reflect how adhering to the JLA guidelines and involving users in different ways impacted personal experiences and the role of the researcher. Later, we delve deeper into these aspects as they emerge from the narratives, situating them within a wider sociocultural and political context, focusing on the ambivalence concerning collaboration with the specific user group in a process of PSP.

### 4.1 Ambivalence Towards Becoming a JLA Researcher—Intrinsic or Extrinsic Motivation and Competing Paradigms

The narratives illuminated contradictory feelings towards following the specific framework outlined in the JLA to identify a knowledge gap and prioritise research needs. The first narrative disclosed insecurity and confusion regarding how to understand and relate to the guidelines of JLA. The process seems to have left the researcher questioning whether her methodological toolbox should be replaced with JLA's PSP in research, as if she were being squeezed between different methodologies or knowledge paradigms. In contrast, the second narrative disclosed reflections about the process of JLA as a much‐needed approach, recognising healthcare professionals' everyday life experiences from clinical practice as an important contribution to the corpus of scientific knowledge production.

The JLA guidelines implied assumptions about who has the most important knowledge. Involving users or the public and questioning how knowledge is produced is not new in international or national (critical) research traditions [27, 28, 29, 41]. There have been strong polarisations of perspectives and paradoxes in the public debate around research needs, user participation and questions about who has knowledge or experiences that qualify as knowledge. These discourses point to a significant mismatch between the research focus and the needs of patients, carers, professionals and healthcare services. According to the JLA, through systematic searches of available knowledge and solid procedures for user involvement, one can help ensure that previously unanswered research questions are relevant and address the needs of patients, carers and clinicians [12]. The recognition and emphasis on experiential medical knowledge have not only raised concerns about scientific rigour but have also been discussed in terms of addressing researchers' concerns about the potential devaluation of scientific knowledge [1, 38, 42, 43]. Do we catch a glimpse of the more old‐fashioned division between the (objective) experiential ‘evidence‐based knowledge’ with randomised‐controlled studies, which has often served as the ‘gold standard’ in biomedicine as opposed to the (subjective) interpretation within social science? [41, 43, 44]. Can it be that the researcher narratives address a tension between different competing scientific knowledge paradigms, which seem to be illuminated within the discussion of wasted research and the JLA when referring to useful methods? Can it be that through the narratives, we spot experiences of struggling with a sense of being undervalued personally and professionally?

Several studies claim that researchers find themselves in difficult and disempowered situations when requirements imposed by health policies and funding institutions mean that excluding users is no longer an option [13, 39, 40, 45]. The aim is to empower marginalised persons by raising consciousness and the status of experience‐based knowledge as a form of bridge building between clinical practice and science. Scholars have expressed scepticism about the prevailing view of researchers, arguing that portraying them as reluctant and the primary source of collaboration challenges might oversimplify the complexity of user involvement in research [40, 46]. Do the narratives raise a concern that the researchers were subjected to somewhat stricter guidelines that overruled them, thus altering their typical role and overriding the qualitative methods that the researchers usually experienced as their area of expertise? Hence, we argue that the JLA guidelines align with the evidence‐based knowledge paradigm in their belief in adhering to a ‘recipe’ or strict methodological criteria for identifying the need for knowledge. This also extends to the perspective on wasted research.

### 4.2 Ambivalence Towards Becoming a Street‐Level Bureaucrat Researcher—Hierarchy and Dual Roles

The two narratives also illuminated other aspects of uncertainty, including ambivalence towards involving professionals who were experts in healthcare and research. The accounts reflected an insecurity in encounters with the user panels, which might have contributed to challenging the researchers' authority in the research process. Questions such as ‘Who is to decide the final research questions, which are also related to the methods of investigation?’ and ‘When or how should user participants be involved in the process?’ arose. According to the researchers' accounts, they feared losing control over the direction of the study. They further revealed confusion about their scientific position and role as researchers within this form of user involvement.

As stated in policy documents and numerous studies, user involvement aims to provide better research and healthcare services [1, 3, 4]. However, various conditions may hinder collaboration, where power struggles between users and researchers have been indicated as one of the main challenges [32, 47]. Following the theory of Lipsky [18], the narratives can be interpreted as expressions of the researchers as the new ‘street‐level bureaucrats'. The lessons learned through this autoethnographic study might be understood as if the researchers experienced becoming a governmental ‘tool’ for policy, helping to implement user involvement through financing studies that use JLA. This might appear to contrast with the ideals usually associated with the assignment, such as being politically free and independent, leading to the ambivalence expressed in the researchers' narratives. Nevertheless, according to Lipsky, frontline providers also have the power—although within restricted frames—to find acceptable solutions for user involvement in research as far as it harmonises with the assignment [18]. In addition, the term users mostly refer to patients or caregivers who have limited power. In our case, the roles were partly reversed—the users were primarily professionals within healthcare and/or research, holding positions equal to or higher than ours. These might be aspects seen within the narratives about user involvement according to the JLA. The researchers navigated their positions within the perceived gap between various ideals and scientific methods and between clinical and researcher knowledge.

The narratives also involved a fear of losing access to the clinical field and a sense of dependence on the goodwill of expert users. Both narratives addressed challenges and dilemmas related to the researchers' positions, highlighting how the JLA PSP made them question their role and identity as researchers with responsibility for the PhD project. Several studies highlight the pressures researchers face, noting that they find themselves in challenging and disempowered situations when compelled by health policies and funding requirements to include users [39, 40, 45]. Boylan et al. showed that researchers felt obligated to be polite and provide emotional support, although they would have preferred to focus on scientific processes [39]. Challenges identified in their study included the dominant positive view of involvement, making it difficult to voice criticism about such practices. Lipsky's theory allowed us to pay attention to the conflicts of self and identity within the researcher's narratives [18]. Komporozos‐Athanasiou, Paylor, and Mckevitt claim that users and researchers find themselves trapped in a system that ‘seeks to reshape their roles and legitimacy’ [40]. Through their qualitative study, these authors observed a degree of researcher reluctance towards the system and illustrated how health policies govern both researchers and their conduct. In other studies, researchers worried about the time commitment and added expenses due to user participation and experienced this as a significant challenge [34, 37, 48]. As a research team, being the ones representing the official guidelines and policy, likely impacted insecurity about one's scientific position and researcher role and the management of these dual roles.

### 4.3 Strengths and Limitations—Sharing (Autoethnographic) Narratives

In this study, we drew on our personal experiences and reflections from the user involvement process of developing research questions for a PhD project. Despite accounts of ambivalence or mixed feelings, identifying and prioritising research needs with users, primarily from clinical practice and research, was also a beneficial and fruitful process for developing topics for research questions. However, the focus has neither been on the outcome of the user involvement process nor the perspective of the involved users. Rather, we aimed at exploring what we can learn from these narratives in a broader sociocultural context. We draw attention to the interface between public guidelines for user involvement and ideals about free research, and the challenges faced by researchers when specific guidelines are required that involve strict procedures.

Using personal experiences and emotions in research has increased considerably over the last decades, particularly in the health sciences [23, 24]. One criticism of autoethnographic studies have been that the narratives often remain within emotional accounts rather than interpreted and discussed from a broader analytical perspective [49]. Chang asks, ‘What drives scholars to expose their personal stories for the academic audience and to allow the public scrutiny of themselves?’ [50]. Some argue that such writing processes might have a therapeutic effect on the authors but are of limited scientific interest if they fail to recognise the personal experiences in light of sociocultural and political contexts [49, 50]. Through personal narratives, we might gain access to what is mostly not accessible [22].

This autoethnographic study originates from researchers having quite different backgrounds and relation to the developed narratives. As authors of this article, we serve as both the research instrument and data, which are typically separated in research. Moreover, much of the external data was produced by us. When dealing with self‐data, which is familiar to oneself, researchers can easily be influenced by their own presumptions about personal experiences without the benefit of fresh perspectives from others who could question the assumptions [22]. CAE attempts to overcome these limitations. Collaborative work allows researchers to benefit simultaneously from self‐ and collective analysis. It has the potential for a deeper understanding of self and others in a sociocultural context, beyond personal feelings of ambivalence.

This retrospective study was developed more than 4 years after the collaboration with the user panels in JLA PSP, which might imply greater distance. The researchers in this study were differently positioned regarding closeness and distance to the narratives. Still, we are the ones who have chosen what to include or exclude and how to tell the stories, and we might have presented the narratives favourably to ourselves. Storytelling can be seen as a performance of self [51]. However, the narratives also illuminate the challenges of meeting contradictory expectations in research, showing the complexity of user involvement in JLA from the researchers' insider and bottom‐up perspectives. Particularly, CAE has been argued that can yield important insights into the research process [52]. By intertwining the two narratives and our interpretation, we can challenge oversimplified perceptions of knowledge, user involvement and the researcher role, thereby offering a more nuanced understanding. Thus, this autoethnographic study can serve as a means of both reflexivity and resistance [25, 44].

## 5 Conclusion and Implications

The narratives identified a struggle to appear as credible researchers with dignity in relation to the user participants, who were primarily professionals with extensive clinical competence. The findings also pointed to the relevance of the sociocultural backdrop; researchers might experience becoming frontline providers of policy implementation, balancing tensions between regulatory constraints, user involvement and their professional identity and research ideals.

As in many countries, the Research Council of Norway require patient and public involvement in research, encouraging to include users in the startup of new projects involving a process of needs‐identified research [1, 3, 5]. Our study highlights certain noteworthy lessons to be learnt. These include challenges faced in the researcher role when guidelines are too inflexible or stringent regarding requirements for how to perform user involvement in research within limited timeframes for PhD projects. This rigidity can potentially hinder both knowledge production in the project and the development of other essential learning skills during a PhD programme. Researchers might become frontline providers of policy implementation, balancing tensions between regulatory constraints, user involvement and researchers' professional role and research ideals. Various kinds of knowledge are needed in different research projects. Different research methods are also required for knowledge production. Therefore, flexibility is required in the approach to user involvement in healthcare research.

## Author Contributions

**Anne Werner:** Conceptualisation, investigation, writing–original draft, methodology, writing–review & editing. **Ingrid Ruud Knutsen:** Methodology, formal analysis, writing–review & editing, conceptualisation. **Anne‐Kari Johannessen:** Writing–original draft, conceptualisation, methodology, writing–review & editing, formal analysis, investigation.

## Ethics Statement

This study followed the Declaration of Helsinki on ethical principles for medical research involving human subjects and was approved by the national IRB (NSD‐472886) and the hospital institutional IRBs (ref. 2020_124, 20/05868).

## Consent

Written informed consent was obtained by the users when they confirmed their participation in the PhD project. In preparing the manuscript, we followed the Consolidated Criteria for Reporting Qualitative Research (COREQ) guidelines for reporting on qualitative research.

## Conflicts of Interest

The authors declare no conflicts of interest.

## Data Availability

The data generated and analysed in this study were developed based on different data sources. The self‐reflective data from recollecting memories of experiences from the user involvement process and the written reports and personal logs from the meetings and seminars are not publicly available from the corresponding authors due to local ownership of data. Aggregated data are available from the corresponding author upon reasonable request.
